# Oral L-carnitine supplementation lowers serum uric acid and is associated with renal and intestinal urate-handling responses in hyperuricemic mice

**DOI:** 10.3389/fnut.2026.1866695

**Published:** 2026-09-15

**Authors:** Fengyu Jin, Yujiao Huang, Fengwei Zhang, Jing He, Jia Zheng, Minghui Jiang, Jiakang Jiao, Yinming Zhao, Jinlian Liu, Jianjun Zhang, Linyuan Wang

**Affiliations:** 1 School of Chinese Materia Medica, Beijing University of Chinese Medicine, Beijing, China; 2 School of Traditional Chinese Medicine, Beijing University of Chinese Medicine, Beijing, China; 3 School of Traditional Chinese Medicine, Inner Mongolia Medical University, Hohhot, China

**Keywords:** ABCG2, gut microbiota, hyperuricemia, L-carnitine, OAT1, urate homeostasis

## Abstract

**Introduction:**

Hyperuricemia (HUA) is a common metabolic disorder associated with gout, renal dysfunction, and intestinal microenvironment disturbance. Impaired urate excretion is considered a major driver of sustained HUA. However, whether nutritional interventions are associated with renal and intestinal urate-handling-related responses remains unclear. This exploratory study investigated whether oral L-carnitine supplementation reduces serum uric acid levels and is accompanied by renal, intestinal, and microbiota-related changes.

**Methods:**

Male C57BL/6 mice were fed a UA/PO-supplemented diet for 12 weeks to establish the HUA model. Mice were assigned to a control group, an untreated HUA group, and L-carnitine-treated groups. Serum uric acid and renal-related biochemical indices were measured. Renal and intestinal histology, urate transporter expression, renal signaling pathways, and gut microbiota composition were evaluated to characterize hepatic urate production-related and renal–intestinal urate-handling-related responses.

**Results:**

Oral L-carnitine supplementation reduced serum uric acid levels and improved renal-related biochemical indices. It did not significantly affect hepatic XOD or ADA, indicating that the urate-lowering effect was not primarily explained by reduced hepatic urate production. In the kidney, L-carnitine restored OAT1 expression and attenuated NF-κB-related inflammatory signaling. In the intestine, it improved villus structure, increased ZO-1 and Occludin expression, and was associated with increased intestinal ABCG2 expression. Gut microbiota structure was also partially restored.

**Conclusion:**

These findings suggest that oral L-carnitine may act as a potential nutritional modulator of urate homeostasis. Its urate-lowering effect was not accompanied by detectable suppression of hepatic urate production, but was associated with renal and intestinal urate-handling-related responses. However, because standardized 24-h urinary and fecal urate excretion measurements and transporter functional assays were not performed, enhanced renal or intestinal urate excretion was not demonstrated.

## 1 Introduction

Hyperuricemia (HUA), characterized by elevated serum uric acid (UA), is a common metabolic disorder and an increasing public health concern (1, 2). Persistent elevation of UA is strongly associated with gout and renal dysfunction, and growing evidence suggests that disturbed urate homeostasis may also contribute to broader metabolic abnormalities (3, 4). With changes in diet and lifestyle, the prevalence of HUA continues to rise, highlighting the need for safe and sustainable nutritional strategies for long-term urate control.

HUA develops when the balance between UA production and UA elimination is disrupted (5, 6). Although excessive UA production can contribute to disease progression, impaired excretion is considered a major determinant of sustained hyperuricemia (7, 8). In this context, the kidney is the principal organ responsible for urate handling, and renal tubular transporters play a central role in determining serum UA levels. Secretory and reabsorptive transport systems jointly regulate renal UA homeostasis, with OAT1 participating in basolateral uptake associated with tubular secretory handling, whereas URAT1 and GLUT9 are more closely related to tubular reabsorption (9, 10). In addition to the kidney, the intestine has emerged as an important extra-renal route for UA elimination (11). The efflux transporter ABCG2, which is highly expressed in the intestine, contributes substantially to enteric urate excretion and may partially compensate when renal urate handling is impaired (12, 13). In humans, approximately 70% of daily uric acid excretion occurs via the kidneys, while the intestinal tract accounts for the remaining 30% (11, 13). Increasing evidence also indicates that HUA is associated with intestinal barrier dysfunction and gut microbiota disturbance (14, 15), suggesting that urate homeostasis is regulated not only by host transport systems but also by the intestinal microenvironment. Although renal and intestinal urate excretion pathways have been widely studied, they are often examined separately, and whether nutritional interventions are associated with concurrent renal and intestinal urate-handling-related responses remains unclear.

Current pharmacological therapy for HUA mainly relies on urate-lowering agents that inhibit UA synthesis or promote its excretion. Although these drugs are effective, their long-term use may be limited in some patients by adverse effects, including hepatic, renal, and gastrointestinal complications (16–18). These limitations have increased interest in dietary compounds that may improve urate homeostasis through nutrition-relevant mechanisms with better long-term tolerability.

L-carnitine is a naturally occurring nutrient derived from lysine and methionine and is widely used as a dietary supplement. Its primary physiological role is to transport long-chain fatty acids into mitochondria for β-oxidation, thereby supporting cellular energy metabolism (19, 20). In addition to this metabolic function, L-carnitine has been reported to exert antioxidant, anti-inflammatory, and tissue-protective effects in different experimental and clinical settings (21–23). These biological properties are relevant to HUA because persistent UA elevation is commonly accompanied by oxidative stress, renal inflammatory injury, mitochondrial metabolic disturbance, and intestinal microenvironment disruption.

Previous studies have suggested that L-carnitine may reduce circulating UA levels, improve renal-related biochemical indices, and alleviate hyperuricemia-associated metabolic and organ injury, partly through regulation of oxidative stress, inflammation, and mitochondrial energy metabolism (24–27). However, most existing studies have focused on general metabolic improvement or tissue protection, while its direct relationship with urate-handling pathways remains insufficiently defined. In particular, whether oral L-carnitine supplementation lowers serum uric acid and is accompanied by renal urate transporter-related changes, intestinal barrier-related responses, and gut microbiota alterations remains insufficiently clarified.

In this study, we used a UA/PO-supplemented diet-induced HUA mouse model to evaluate the effects of oral L-carnitine supplementation, with a focus on hepatic urate production-related indices, renal and intestinal urate transporter expression, intestinal barrier-related changes, renal signaling pathways, and gut microbiota alterations. We aimed to determine whether the urate-lowering effect of oral L-carnitine was associated with renal and intestinal urate-handling-related responses rather than detectable suppression of hepatic urate production.

## 2 Materials and methods

### 2.1 Materials and reagents

L-carnitine (purity >98%) and potassium oxonate (purity >98%) were purchased from Shanghai Yuanye Bio-Technology Co., Ltd. (Shanghai, China). Uric acid (purity >98%) was purchased from Chengdu DeSiTe Biological Technology Co., Ltd. (Chengdu, China). Allopurinol tablets were manufactured by Shanghai Xinyi Wanxiang Pharmaceutical Co., Ltd. (Shanghai, China; batch No. 43220704). All interventions were freshly prepared in water as the vehicle before oral gavage. The uric acid assay kit was purchased from China Gate Beijing Control Bio-Technology Co., Ltd. (Beijing, China). Other biochemical assay kits and reagents are described in the corresponding methods.

### 2.2 Animals and experimental design

Specific pathogen-free (SPF) male C57BL/6 mice (6–8 weeks old) were obtained from Beijing Weitong Lihua Experimental Animal Technology Co., Ltd. (Beijing, China). Male mice were used to reduce potential variability associated with the estrous cycle, although this sex restriction may limit the generalizability of the findings. Mice were housed at the Laboratory Animal Center of Beijing University of Chinese Medicine under standard SPF conditions (25 ± 1 °C, 50 ± 1% humidity, and a 12-h light/dark cycle) with ad libitum access to food and water. Animals were housed at three or four mice per standard cage with corncob bedding. General health status, body weight, food intake, fur condition, posture, locomotor activity, and signs of distress were monitored throughout the experiment.

All experimental procedures were approved by the Medical Laboratory Animal Ethics Committee of Beijing University of Chinese Medicine (Approval No. BUCM-2024030506-1134) and were conducted in accordance with institutional animal care guidelines. After one week of acclimatization, 35 mice were numbered and randomly allocated into five groups using a random number-based method, with seven mice per group: normal control group (CG), hyperuricemia model group (HUG), allopurinol group (AG), low-dose L-carnitine group (LLG), and high-dose L-carnitine group (HLG). The individual animal was defined as the experimental unit for *in vivo* biochemical, histological, and molecular analyses.

To establish the hyperuricemia model, all groups except the CG were fed a UA/PO-supplemented diet for 6 weeks, while the CG received a standard diet. At week 6, serum UA levels were measured to confirm successful model establishment at the group level. During the subsequent 6-week intervention period, mice continued on their respective diets and received daily oral gavage rather than feed-incorporated supplementation. The AG received allopurinol at 5 mg/kg, the LLG received L-carnitine at 50 mg/kg, and the HLG received L-carnitine at 100 mg/kg. The CG and HUG received an equivalent volume of water vehicle. All interventions were freshly prepared in water before administration and were administered once daily at a gavage volume of 10 mL/kg before routine daily food replenishment. Mice were not subjected to a separate fasting procedure before gavage. The L-carnitine doses were selected based on previous rodent studies using comparable or higher oral supplementation doses and available safety evidence (28–31). Allopurinol was included as a classical urate-lowering reference drug to assess the responsiveness of the HUA model, rather than as a mechanism-matched uricosuric comparator.

Because different interventions were administered by oral gavage, complete blinding of treatment administration was not feasible. However, sample labels were coded before downstream analyses whenever feasible, and standardized procedures were applied across groups to minimize potential assessment and procedural bias.

Inclusion criteria included successful completion of acclimatization, absence of overt disease or injury before modeling, and completion of the experimental protocol. Humane or exclusion criteria included severe weight loss, persistent inability to access food or water, severe lethargy, abnormal posture, marked dehydration, severe injury, or death unrelated to the experimental intervention. No animals died during the experiment, and no animals or data points were excluded from the main efficacy analysis.

The main *in vivo* efficacy experiment included seven mice per group. For exploratory downstream analyses, including Western blotting, immunofluorescence, transcriptomic profiling, and 16S rRNA gene sequencing, three or four independent biological replicates per group were used depending on tissue availability, sample quality, and assay requirements. Each biological replicate represented one individual animal.

### 2.3 Preparation of hyperuricemia-inducing diets

The basal diet was standard mouse maintenance chow purchased from Keao Xieli Feed Co., Ltd. (Tianjin, China; batch No. 24033213). According to the manufacturer-provided nutritional information, the basal diet contained moisture ≤10%, crude protein ≥18%, crude fat ≥4%, crude fiber ≤5%, crude ash ≤8%, calcium 1.0–1.8%, and total phosphorus 0.6–1.2%. The energy density of the basal diet was 3.40 kcal/g, with energy contributions of 23.07% from protein, 11.85% from fat, and 65.08% from carbohydrates.

The hyperuricemia-inducing diet was prepared by supplementing the basal diet with 2% potassium oxonate and 3% uric acid (w/w). The customized feed was prepared by Keao Xieli Feed Co., Ltd. (Tianjin, China) and sterilized by gamma irradiation before use.

### 2.4 Biochemical analyses

To evaluate the urate-lowering efficacy of L-carnitine, serum uric acid (UA) levels were quantified. Renal function was assessed by measuring serum creatinine (CREA) and urea levels using an automated biochemical analyzer, and the urea-to-creatinine ratio was subsequently calculated. To provide additional intestinal-side information related to urate handling, endpoint fecal UA content was measured. Briefly, 0.05 g of freshly collected feces was accurately weighed and homogenized in 0.20 mL of saline to prepare a 20% (w/v) fecal homogenate. The homogenates were centrifuged at 1,000 × g for 10 min at 4 °C, and the resulting supernatants were collected for UA determination using a commercial UA assay kit. In addition, hepatic xanthine oxidase (XOD) and adenosine deaminase (ADA) activities were measured to evaluate changes associated with hepatic urate production. All biochemical assays were performed strictly according to the manufacturers’ protocols.

### 2.5 Histopathological examination

To evaluate histopathological alterations, samples of the liver, kidney, and small intestine were harvested and immediately fixed in 4% paraformaldehyde. Following standard dehydration and paraffin embedding, the tissues were cut into 5-μm-thick sections and subsequently stained with hematoxylin and eosin (H&E). Morphological changes were then examined and photographed under a light microscope.

### 2.6 Western blotting

Total proteins from the target tissues were extracted using RIPA lysis buffer supplemented with a protease inhibitor cocktail. Following centrifugation, protein concentrations were determined using a BCA protein assay kit. Equal amounts of protein samples were separated by SDS-PAGE and transferred onto PVDF membranes. After blocking with 5% non-fat milk in Tris-buffered saline containing 0.1% Tween 20 (TBST) for 1 h at room temperature, the membranes were incubated overnight at 4 °C with the following primary antibodies: XOD (1:2000, Cat. No. 55156-1-AP, Proteintech), ADA (1:2000, Cat. No. 67870-1-Ig, Proteintech), OAT1 (1:2000, Cat. No. 26574-1-AP, Proteintech), OAT3 (1:2000, Cat. No. bs-0609R, Bioss), GLUT9 (1:1000, Cat. No. 26486-1-AP, Proteintech), ABCG2 (1:1000, Cat. No. 27286-1-AP, Proteintech), URAT1 (1:3000, Cat. No. 14937-1-AP, Proteintech), IκBα (1:1000, Cat. No. GB111509, Servicebio), NF-κB p65 (1:2000, Cat. No. 66535-1-Ig, Proteintech), phospho-NF-κB p65 (1:2000, Cat. No. ZHS1565, ZhongHe) and β-actin (1:8000, Cat. No. 66009-1-Ig, Proteintech). All primary antibodies were diluted in a universal antibody diluent.

After three washes with TBST for 10 min each, the membranes were incubated with HRP-conjugated goat anti-rabbit IgG (1:2000, Cat. No. GB23303, Servicebio) or HRP-conjugated goat anti-mouse IgG (1:8000, Cat. No. GB23301, Servicebio) for 1 h at room temperature. Immunoreactive bands were visualized using an enhanced chemiluminescence system and semi-quantitatively analyzed using ImageJ software. Total proteins were normalized to β-actin, whereas phosphorylated proteins were normalized to their corresponding total proteins.

### 2.7 Immunofluorescence staining

Following deparaffinization and rehydration, the tissue sections were subjected to heat-induced antigen retrieval in a standard retrieval buffer. The sections were then permeabilized with 0.5% Triton X-100 in PBS and blocked with 5% BSA at room temperature to prevent non-specific binding. Subsequently, the tissues were incubated overnight at 4 °C with primary antibodies targeting OAT1 (Cat. No. 26574-1-AP, Proteintech), ABCG2 (Cat. No. 27286-1-AP, Proteintech), ZO-1 (Cat. No. GB111981, Servicebio), Occludin (Cat. No. GB111401, Servicebio) and NF-κB p65 (Cat. No. 66535-1-Ig, Proteintech) (all diluted at 1:200). After stringent washing with PBS, the sections were incubated with appropriate fluorophore-conjugated secondary antibodies for 1 h at room temperature in the dark. Finally, cell nuclei were counterstained with DAPI, and the slides were mounted for fluorescence microscopy visualization.

### 2.8 Transcriptomic profiling

For exploratory renal transcriptomic profiling, kidney tissues from the CG, HUG, and HLG groups were used, with four independent biological replicates per group. These groups were selected to compare the normal condition, the hyperuricemic state, and the high-dose L-carnitine intervention group.

Total RNA was extracted from mouse kidney tissues using TRIzol reagent according to the manufacturer’s instructions. RNA concentration and purity were determined using a NanoDrop 2000 spectrophotometer, and RNA integrity was assessed using an Agilent 2100/5400 Bioanalyzer to ensure that samples met the criteria for library construction. Poly(A) mRNA was enriched from total RNA using oligo(dT) magnetic beads. The purified mRNA was fragmented under controlled conditions and used as a template for first-strand cDNA synthesis with random hexamer primers and reverse transcriptase. Second-strand cDNA was then synthesized using DNA Polymerase I and dNTPs. The resulting double-stranded cDNA was purified using AMPure XP beads, followed by end repair, 3′ adenylation, adapter ligation, library construction, and sequencing.

### 2.9 16S rRNA gene sequencing

For exploratory 16S rRNA gene sequencing, fecal samples from the CG, HUG, and HLG groups were collected, with four independent biological replicates per group. These groups were selected to compare the normal condition, the hyperuricemic state, and the high-dose L-carnitine intervention group.

Total genomic DNA was extracted using a commercial fecal DNA kit. The integrity of the extracted DNA was verified by 1% agarose gel electrophoresis, and DNA concentration and purity were determined using a micro-spectrophotometer. The hypervariable regions of the bacterial 16S rRNA gene were amplified by PCR using primers with unique barcodes. The PCR products were purified and used for sequencing library construction. High-throughput sequencing was performed on the Illumina MiSeq PE250 platform in a paired-end format.

Alpha diversity indices were calculated to assess microbial richness and evenness within samples. Beta diversity was evaluated based on distance matrices and visualized using principal coordinate analysis (PCoA) to illustrate differences in microbial community structure among groups. Statistical significance was assessed using appropriate non-parametric tests.

### 2.10 Molecular docking analysis

Molecular docking was performed to explore the potential interaction between L-carnitine and RELA/p65. The three-dimensional structure of L-carnitine was obtained from PubChem and energy-minimized using ChemBio3D Ultra 14.0. The crystal structure of RELA (PDB ID: 2O61) was obtained from the Protein Data Bank and prepared using PyMOL 2.3.0 and AutoDockTools 1.5.6. Molecular docking was conducted using AutoDock Vina 1.1.2, and the binding pose with the lowest binding energy was selected for interaction analysis and visualization in PyMOL.

### 2.11 Statistical analysis

All data are presented as the mean ± standard error of the mean (SEM). Longitudinal body weight data were analyzed using two-way repeated-measures ANOVA, with group and time as factors. When sphericity could not be assumed, the Geisser–Greenhouse correction was applied. No missing values were present for the longitudinal body weight analysis. Endpoint data, including serum uric acid levels after the 6-week intervention, were first assessed for normality and variance homogeneity and then analyzed using one-way ANOVA followed by Tukey’s multiple-comparison test when assumptions were met. When assumptions were not met, appropriate nonparametric tests were used. Statistical analyses and graphical representations were performed using GraphPad Prism 8.0.2. A *p*-value < 0.05 was considered statistically significant.

## 3 Results

### 3.1 L-carnitine lowered serum uric acid and improved renal-related biochemical indices in hyperuricemic mice

To evaluate the effect of oral L-carnitine on HUA, mice were fed a UA/PO-supplemented diet for 12 weeks (Figure 1A). No significant differences were observed in body weight gain (Figure 1B), and no obvious changes in fur condition were noted among the groups, suggesting that L-carnitine treatment did not markedly affect overall growth under the present experimental conditions.

**Figure 1:**
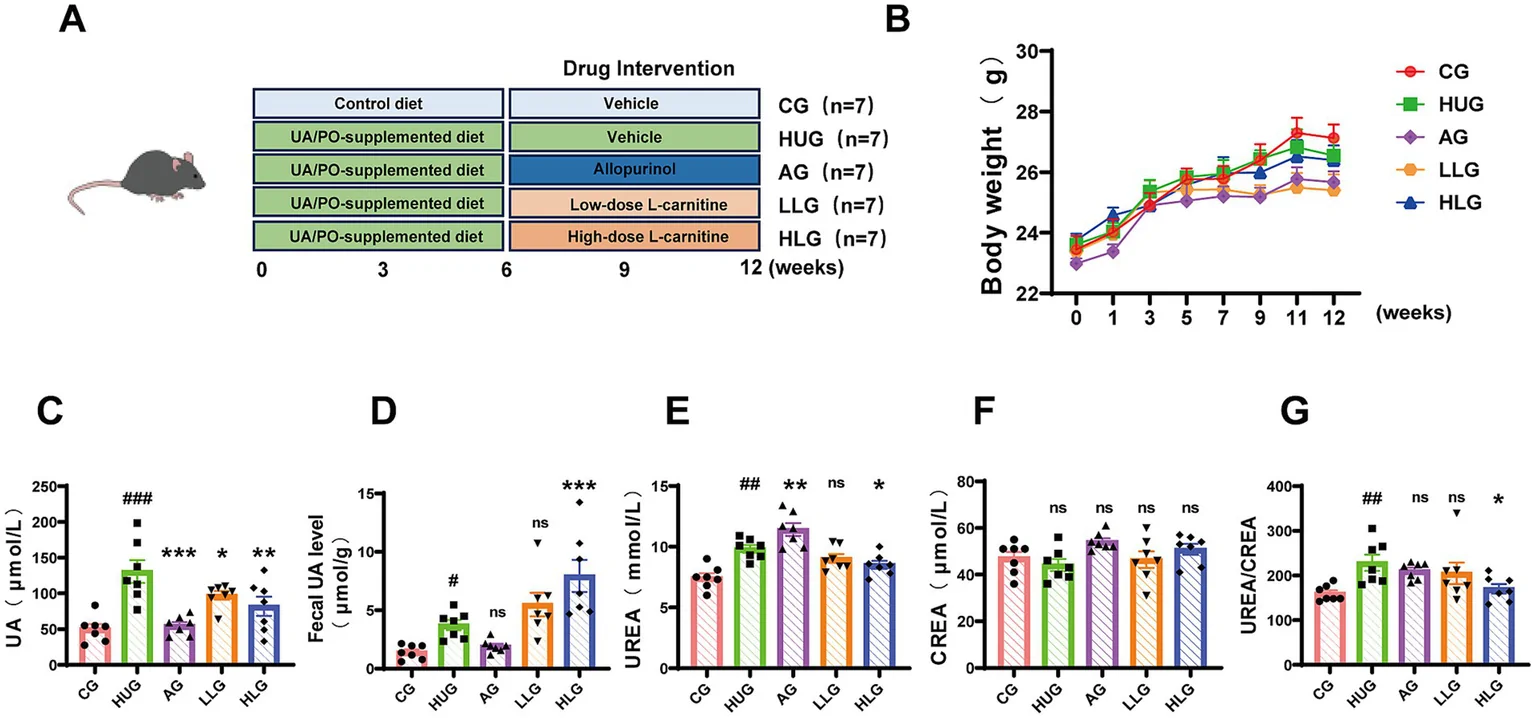
Effects of L-carnitine on uric acid and renal-related biochemical indices in hyperuricemic mice. **(A)** Experimental design. **(B)** Body weight changes during the 12-week experimental period. **(C)** Serum UA levels. **(D)** Fecal UA content. **(E)** Serum urea levels. **(F)** Serum creatinine (CREA) levels. **(G)** Urea-to-creatinine (UREA/CREA) ratio. Data are presented as mean ± SEM (*n* = 7 per group). ns, not significant; ^#^*p* < 0.05, ^##^*p* < 0.01, ^###^*p* < 0.001 vs. CG; ^*^p < 0.05, ^**^p < 0.01, ^***^p < 0.001 vs. HUG.

At the end of the intervention period, serum UA levels were significantly elevated in the HUA model group compared with the control group. As expected, allopurinol markedly reduced endpoint serum UA levels, confirming the responsiveness of the HUA model to a classical urate-lowering reference drug. Oral L-carnitine supplementation also reduced endpoint serum UA levels compared with the untreated model group, with reductions of 25.4 and 37.2% in the low- and high-dose groups, respectively (Figure 1C). The percentage reduction was calculated relative to the HUG group as: [(mean UA in HUG − mean UA in treatment group) / mean UA in HUG] × 100%.

Endpoint fecal UA content was also measured to provide additional intestinal-side information related to urate handling. Compared with the HUG group, high-dose L-carnitine significantly increased endpoint fecal UA content (Figure 1D). Renal-related biochemical indices showed a different pattern from serum UA. Compared with the untreated model group, high-dose L-carnitine significantly reduced serum urea levels and the urea-to-creatinine ratio (Figures 1E–G). In contrast, although allopurinol markedly reduced endpoint serum UA levels, confirming its effect as a classical urate-lowering reference drug, it did not show the same renal-related biochemical improvement under the present experimental conditions, and serum urea remained elevated in the allopurinol-treated group. These findings suggest that L-carnitine lowered serum UA and was associated with improvement in renal-related biochemical indices in hyperuricemic mice.

### 3.2 L-carnitine lowered serum uric acid without detectable inhibition of hepatic urate production

Histological examination showed that hepatic architecture remained largely intact across all groups, with no evident pathological lesions (Figure 2A). Consistent with this finding, hepatic triglyceride levels did not differ significantly among the groups (Figure 2B). In contrast, hepatic SOD activity was significantly decreased in the model group, whereas high-dose L-carnitine treatment significantly increased SOD activity compared with the untreated HUA group (Figure 2E).

**Figure 2:**
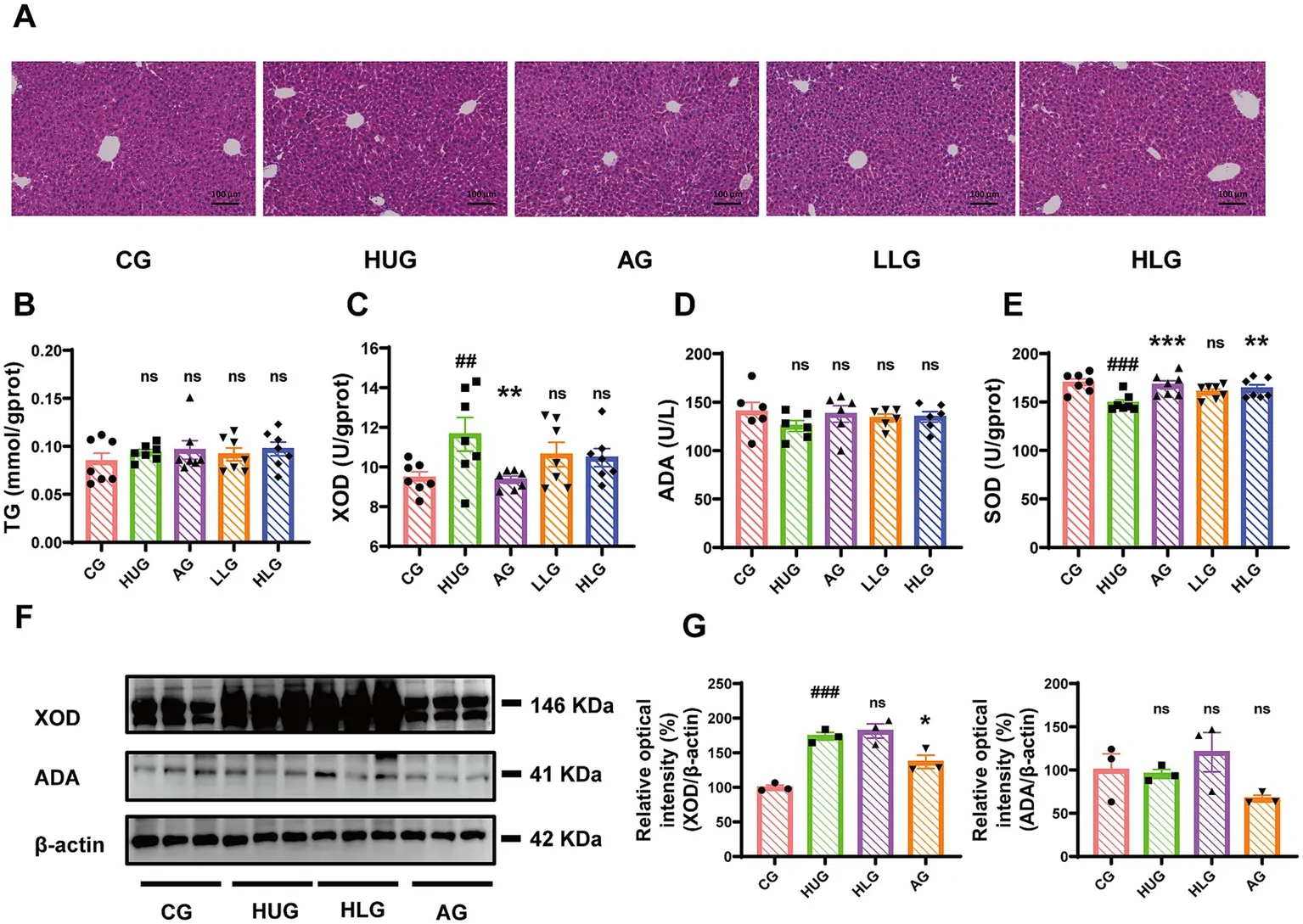
Effects of L-carnitine on hepatic histology, biochemical indices, and urate-producing enzymes in hyperuricemic mice. **(A)** H&E staining of liver sections. **(B)** Hepatic triglyceride levels. **(C)** Hepatic XOD activity (*n* = 7 per group). **(D)** Hepatic ADA activity (*n* = 7 per group). **(E)** Hepatic SOD activity (*n* = 7 per group). **(F)** Representative protein expression of XOD and ADA. **(G)** Quantification of XOD and ADA protein expression (*n* = 3 per group). Data are presented as mean ± SEM. ns, not significant; ^#^*p* < 0.05, ^##^*p* < 0.01, ^###^*p* < 0.001 vs. CG; ^*^p < 0.05, ^**^p < 0.01, ^***^p < 0.001 vs. HUG.

To further assess whether the urate-lowering effect of L-carnitine was related to hepatic urate synthesis, the activities and expression levels of the key urate-producing enzymes xanthine oxidase (XOD) and adenosine deaminase (ADA) were measured. L-carnitine treatment did not significantly affect either the enzymatic activities or the protein expression levels of XOD and ADA (Figures 2C,D,F,G). These results suggest that the urate-lowering effect of L-carnitine in this model was not primarily explained by reduced hepatic urate production.

### 3.3 L-carnitine was associated with recovery of renal OAT1 expression in hyperuricemic mice

H&E staining showed that the overall renal architecture was largely preserved across groups. Compared with the control group, the HUA model group showed mild tubulointerstitial alterations, including renal tubular epithelial swelling, mild tubular luminal dilation, and limited peritubular inflammatory cell infiltration. In the representative images, black arrows indicate renal tubular epithelial swelling, whereas red arrows indicate peritubular inflammatory cell infiltration. L-carnitine supplementation partially ameliorated these histopathological alterations, as evidenced by better preservation of tubular epithelial morphology and reduced peritubular inflammatory cell infiltration (Figure 3A). To further assess renal urate handling, key transporters involved in tubular urate secretion and reabsorption were analyzed.

**Figure 3:**
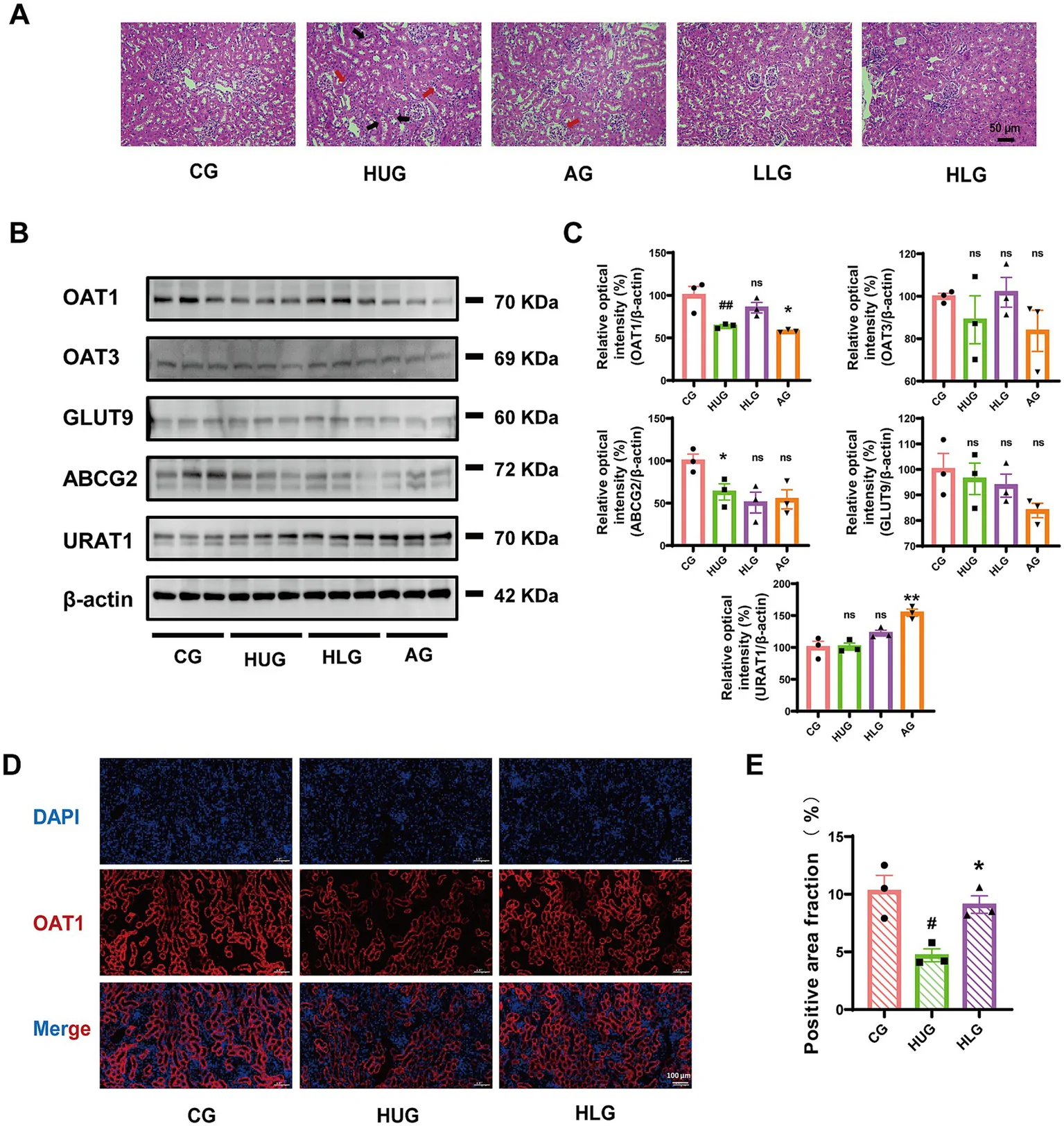
Effects of L-carnitine on renal histology and urate transporter expression in hyperuricemic mice. **(A)** H&E staining of kidney tissue sections. **(B)** Representative protein expression of renal urate transport-related proteins. **(C)** Quantitative analysis of renal urate transport-related protein expression (*n* = 3 per group). **(D)** Immunofluorescence staining of OAT1 in renal tissues. **(E)** Quantification of OAT1 fluorescence intensity. Data are presented as mean ± SEM (*n* = 3 per group). ns, not significant; ^#^*p* < 0.05, ^##^*p* < 0.01, ^###^*p* < 0.001 vs. CG; ^*^p < 0.05, ^**^p < 0.01, ^***^p < 0.001 vs. HUG.

OAT1 protein expression was significantly decreased in the HUG group compared with the CG group, and high-dose L-carnitine treatment significantly restored OAT1 expression (Figures 3B,C). Consistently, renal OAT1 immunofluorescence showed recovery of OAT1 signal after L-carnitine treatment (Figures 3D,E). By contrast, OAT3, GLUT9, and renal ABCG2 did not show significant model-related alterations or consistent L-carnitine-induced changes (Figures 3B,C). URAT1 expression was significantly increased in the allopurinol-treated group, whereas L-carnitine did not consistently reduce URAT1 expression. Because allopurinol is a xanthine oxidase inhibitor rather than a uricosuric agent, the URAT1 increase in the AG group was interpreted cautiously as a treatment-associated transporter response rather than evidence of uricosuric activity.

Overall, OAT1 was the transporter most clearly responsive to HUA modeling and L-carnitine intervention. These findings suggest that L-carnitine-associated urate lowering was accompanied by an OAT1-related renal transporter response. However, because 24-h urinary urate, urinary creatinine, urate clearance, fractional urate excretion, transporter activity, membrane localization, and inhibitor-based functional assays were not performed, these data do not demonstrate enhanced renal urate excretion.

### 3.4 Renal transcriptomic analysis linked L-carnitine treatment to inflammation-related pathways

To investigate the renal molecular changes associated with L-carnitine treatment, RNA-seq was performed using kidney tissues from the CG, HUG, and HLG groups. Differentially expressed genes (DEGs) were identified using the criteria of *p* < 0.05 and |log2(fold change)| > 1. Compared with the CG, 336 DEGs were identified in the HUG, including 194 upregulated and 142 downregulated genes. Compared with the HUG, 398 DEGs were identified in the HLG, including 122 upregulated and 276 downregulated genes. Venn diagram analysis identified 57 overlapping DEGs that were altered in the disease state and responsive to L-carnitine treatment (Figures 4A–D).

**Figure 4:**
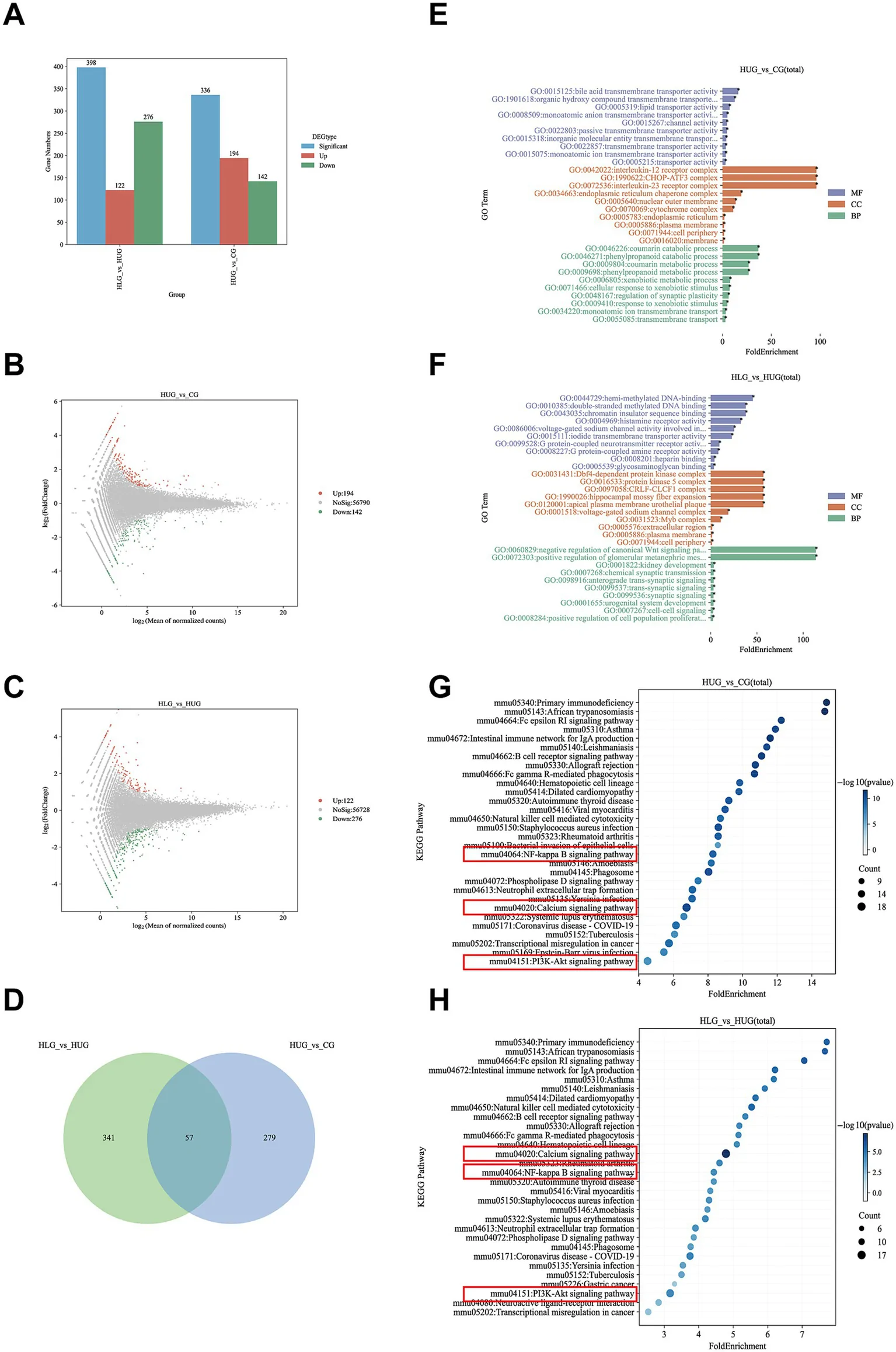
Transcriptomic analysis of renal tissues in hyperuricemic mice following L-carnitine intervention. **(A)** Summary of differentially expressed genes (DEGs) across different comparisons, showing the number of upregulated and downregulated genes. **(B,C)** Volcano plots illustrating the distribution of DEGs in the HUG vs. CG **(B)** and HLG vs. HUG **(C)** comparisons. **(D)** Venn diagram showing the overlapping DEGs between disease-responsive and drug-regulated gene sets. **(E,F)** Gene Ontology (GO) enrichment analysis of DEGs. **(G,H)** KEGG pathway enrichment analysis of DEGs shown in bubble plots (*n* = 4 per group).

To further characterize the functional features of these transcriptomic changes, Gene Ontology (GO) enrichment analysis was performed. In the HUG vs. CG comparison, DEGs were mainly enriched in transmembrane transport-related processes, including bile acid transporter activity and passive transmembrane transporter activity. In the HLG vs. HUG comparison, DEGs were enriched in terms including negative regulation of canonical Wnt signaling pathway and positive regulation of glomerular metanephric mesenchyme development (Figures 4E,F).

KEGG pathway analysis showed that DEGs in the HUG vs. CG comparison were significantly enriched in several signaling pathways, including NF-κB, PI3K-Akt, and calcium signaling pathways. DEGs in the HLG vs. HUG comparison were also enriched in related inflammatory and stress-response pathways (Figures 4G,H). These transcriptomic data indicate that L-carnitine treatment was associated with renal molecular changes involving inflammation-related signaling and transport-related biological processes. Given its relevance to renal inflammatory injury, NF-κB signaling was selected for subsequent protein-level validation. Other enriched pathways, including PI3K-Akt and calcium signaling, were interpreted as exploratory transcriptomic findings requiring further validation.

### 3.5 L-carnitine attenuated renal NF-κB-related inflammatory signaling in hyperuricemic mice

Among the enriched pathways, NF-κB signaling was selected for validation because it was identified by renal transcriptomic analysis and is closely associated with renal inflammation, tubular dysfunction, and stress responses under hyperuricemic conditions. We then examined renal IκBα, p-p65, and p65 protein levels. HUA mice showed an increased p-p65/p65 ratio and reduced IκBα expression compared with the control group (Figures 5A,B), consistent with activation of renal NF-κB signaling under hyperuricemic conditions. L-carnitine treatment reduced the p-p65/p65 ratio and restored IκBα expression, suggesting attenuation of NF-κB-related inflammatory signaling.

**Figure 5:**
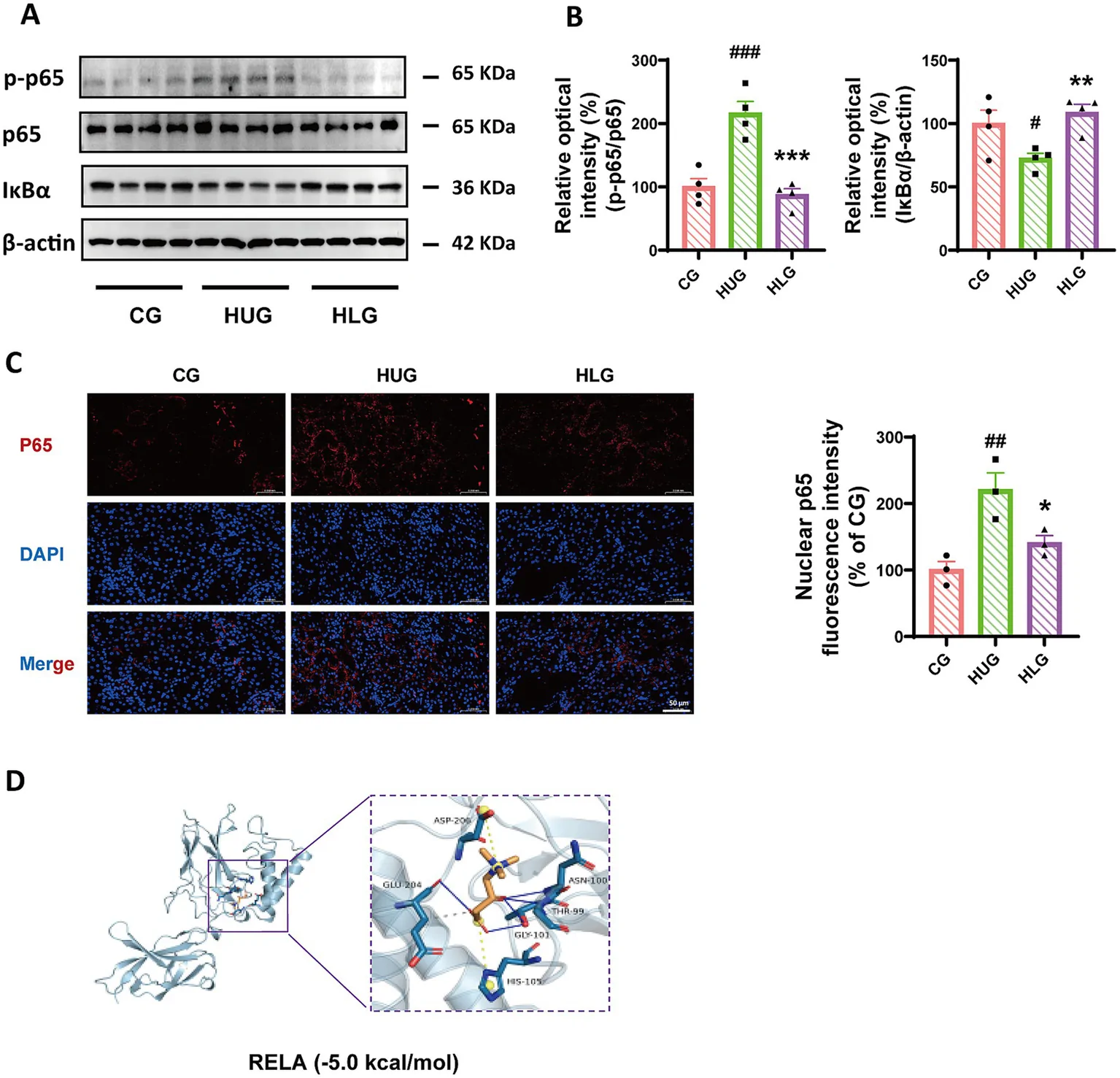
Effects of L-carnitine on renal NF-κB signaling in hyperuricemic mice. **(A)** Representative Western blot bands of IκBα, p-p65 and p65 in kidney tissues. **(B)** Quantitative analysis of IκBα expression and the p-p65/p65 ratios. **(C)** Immunofluorescence staining of p65 in renal sections, showing nuclear translocation of p65. **(D)** Molecular docking simulation of L-carnitine with RELA (p65), with the predicted binding energy. Data are presented as mean ± SEM. Each biological replicate represents kidney tissue from one individual mouse. ^#^*p* < 0.05, ^##^*p* < 0.01, ^###^*p* < 0.001 vs. CG; ^*^p < 0.05, ^**^p < 0.01, ^***^p < 0.001 vs. HUG.

Immunofluorescence staining further showed enhanced nuclear accumulation of p65 in the kidneys of HUA mice, whereas L-carnitine treatment reduced p65 nuclear translocation (Figure 5C). To provide exploratory computational context for the NF-κB-related findings, molecular docking was performed between L-carnitine and RELA/p65. The docking analysis showed a predicted binding energy of −5.0 kcal/mol for L-carnitine with RELA (Figure 5D). However, this in silico result was interpreted only as hypothesis-generating computational evidence and was not used as proof of direct target engagement *in vivo*. Together, these findings suggest that L-carnitine treatment was associated with attenuation of renal NF-κB-related inflammatory signaling in HUA mice.

### 3.6 L-carnitine improved intestinal barrier-related features and increased intestinal ABCG2 expression

H&E staining showed that the overall intestinal mucosal architecture was largely preserved across groups. Compared with the control group, the HUA model group exhibited mild intestinal mucosal injury, mainly characterized by villus morphological damage and inflammatory cell infiltration in the lamina propria. In the representative images, black arrows indicate villus morphological damage, whereas red arrows indicate inflammatory cell infiltration. L-carnitine supplementation partially alleviated these intestinal histopathological alterations, as evidenced by improved villus morphology and reduced inflammatory cell infiltration (Figure 6A). Immunofluorescence staining further showed increased expression of the barrier-related proteins ZO-1 and Occludin in the small intestine after L-carnitine treatment (Figure 6B), suggesting improvement of intestinal epithelial barrier-related features.

**Figure 6:**
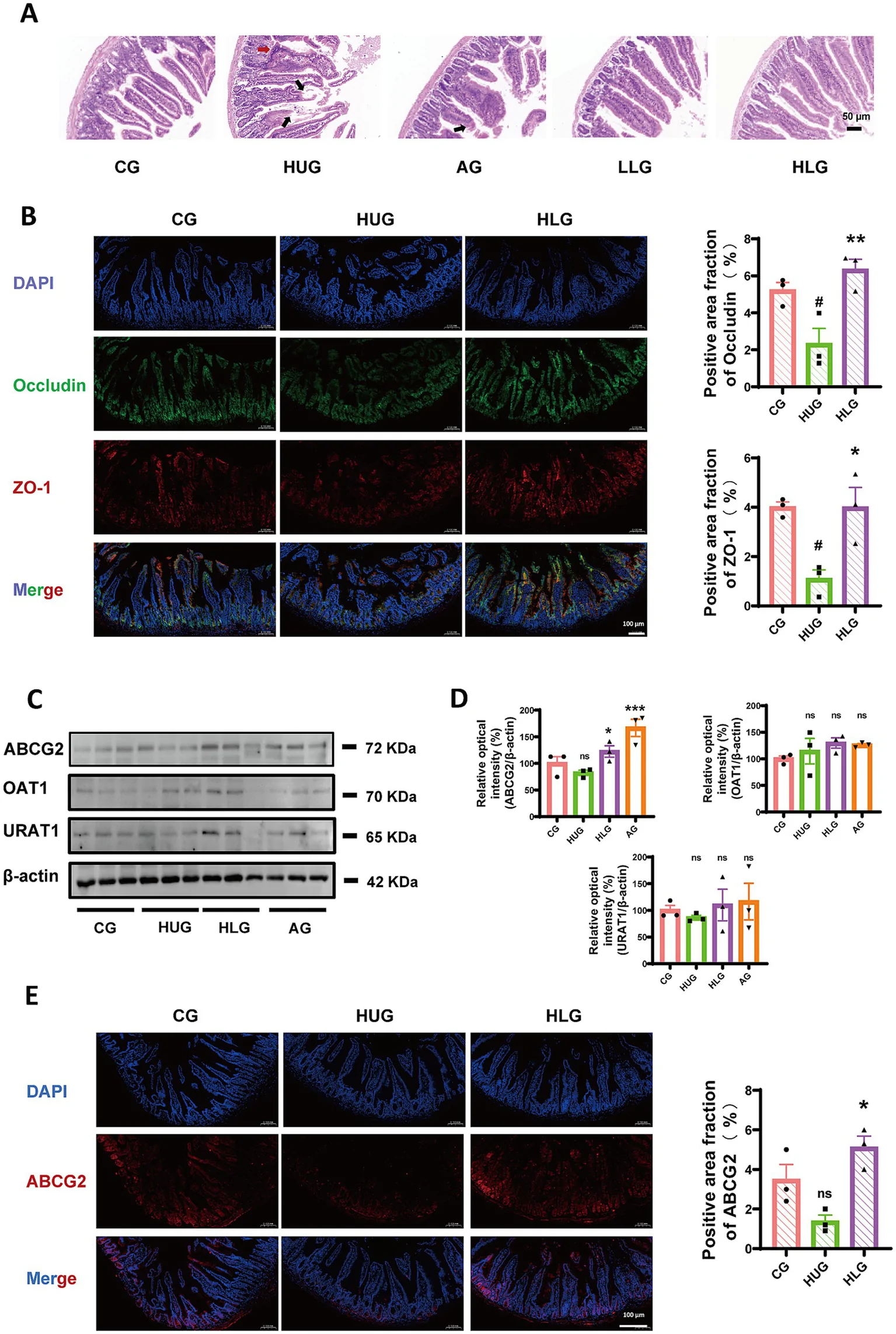
Effects of L-carnitine on intestinal morphology, barrier-related proteins, and urate transporter expression in hyperuricemic mice. **(A)** H&E staining of intestinal tissue sections. **(B)** Immunofluorescence staining of Occludin and ZO-1 in the small intestine (*n* = 3 per group). **(C)** Representative protein expression of intestinal urate transport-related proteins. **(D)** Quantitative analysis of intestinal urate transport-related protein expression (*n* = 3 per group). **(E)** Immunofluorescence staining of ABCG2 in the small intestine (*n* = 3 per group). Data are presented as mean ± SEM. ns, not significant; ^#^*p* < 0.05, ^##^*p* < 0.01, ^###^*p* < 0.001 vs. CG; ^*^p < 0.05, ^**^p < 0.01, ^***^p < 0.001 vs. HUG.

To further assess intestinal urate-handling-related changes, intestinal urate transporter expression was examined, with a focus on ABCG2. Intestinal ABCG2 protein expression did not differ significantly between the CG and HUG groups, indicating that ABCG2 did not show a clear model-related alteration under the present experimental conditions. However, compared with the HUG group, L-carnitine treatment, particularly in the HLG group, increased intestinal ABCG2 protein expression (Figures 6C,D). This finding was supported by ABCG2 immunofluorescence staining, which showed enhanced ABCG2 signal along the intestinal epithelial region after L-carnitine treatment (Figure 6E). Together, these results suggest that L-carnitine was associated with intestinal barrier-related improvement and ABCG2-related urate-handling changes in HUA mice. The increase in endpoint fecal UA content was directionally consistent with the ABCG2-related intestinal response.

### 3.7 L-carnitine partially restored gut microbiota structure in hyperuricemic mice

To explore gut microbiota changes associated with high-dose L-carnitine treatment, 16S rRNA gene sequencing was performed using fecal samples from the CG, HUG, and HLG groups, with four independent biological replicates per group. Alpha diversity analysis showed that microbial richness, evenness, and overall diversity were significantly reduced in the HUA model group compared with the control group (Figures 7A,B). L-carnitine treatment partially restored these indices. Furthermore, principal coordinates analysis (PCoA) based on beta diversity showed clear separation among the groups. Compared with the HUA model group, the high-dose L-carnitine-treated group shifted closer to the control group, indicating partial restoration of the overall gut microbial community structure (Figure 7C).

**Figure 7:**
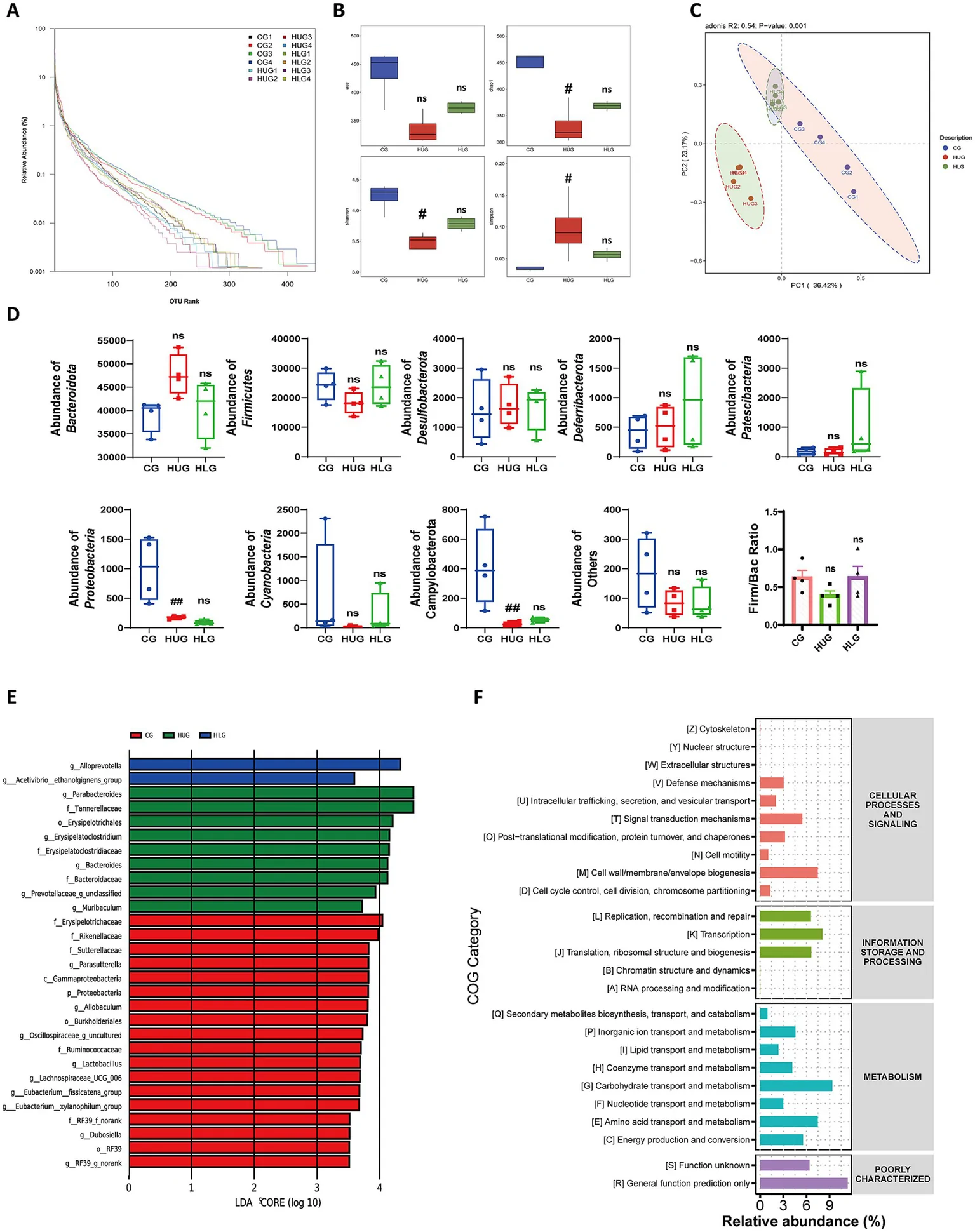
Effects of L-carnitine on gut microbiota structure and predicted functional profiles in hyperuricemic mice. **(A)** Rank-abundance curves. **(B)** Alpha diversity indices (ACE, Chao1, Shannon, and Simpson). **(C)** PCoA based on beta diversity. **(D)** Relative abundance at the phylum level. **(E)** LEfSe analysis of differential taxa. **(F)** Predicted functional profiling based on the COG database. Data are presented as mean ± SEM (*n* = 4 per group). ns, not significant; ^#^*p* < 0.05, ^##^*p* < 0.01 vs. CG.

At the phylum level, the model group showed a decreased relative abundance of Firmicutes and an increased relative abundance of Bacteroidetes compared with the control group, resulting in a reduced Firmicutes/Bacteroidetes ratio (Figure 7D). L-carnitine treatment partially reversed these changes.

LEfSe analysis was performed to identify differential microbial taxa among the groups (Figure 7E). The control group was characterized by 18 discriminative taxa, including *Erysipelotrichaceae* and *Rikenellaceae*, whereas the model group was enriched in *Parabacteroides* and *Tannerellaceae*. After L-carnitine treatment, *Alloprevotella* and the *Acetivibrio ethanolgignens group* were identified as the predominant discriminative taxa.

Predicted functional profiling based on the Clusters of Orthologous Groups (COG) database identified 25 annotated functional categories (Figure 7F). Among these, pathways related to cell wall/membrane/envelope biogenesis, carbohydrate transport and metabolism, and amino acid transport and metabolism were prominently represented. These findings indicate that L-carnitine treatment was associated with partial restoration of gut microbiota structure in hyperuricemic mice.

## 4 Discussion

The present study showed that oral L-carnitine supplementation lowered serum uric acid in UA/PO-supplemented diet-induced hyperuricemic mice and was accompanied by renal and intestinal urate-handling-related responses. In the high-dose group, the reduction in serum UA was also accompanied by lower serum urea and a reduced urea-to-creatinine ratio, indicating concurrent favorable changes in renal-related biochemical indices (32, 33). From a nutritional perspective, these multi-organ responses suggest that L-carnitine may act as a modulator of urate homeostasis rather than as a conventional urate-lowering agent directed primarily at urate production.

Notably, L-carnitine did not significantly alter hepatic XOD or ADA activity or protein expression, while liver histology remained largely unchanged. Thus, the observed reduction in serum UA was not accompanied by detectable suppression of the hepatic urate-production-related pathways examined in this study. This response differs from conventional urate-lowering strategies that primarily inhibit urate synthesis (34, 35) and provides a rationale for focusing subsequent interpretation on the renal and intestinal urate-handling-related changes observed after L-carnitine supplementation.

The kidney appears to be a major site of this response. Hyperuricemic mice showed altered renal-related biochemical indices, mild renal morphological changes, and reduced OAT1 expression. L-carnitine improved renal morphology and restored OAT1 expression, whereas OAT3, GLUT9, and renal ABCG2 showed no clear model-related alterations or consistent L-carnitine-induced changes. URAT1 expression was significantly increased in the allopurinol-treated group, but L-carnitine did not consistently reduce URAT1 expression. Therefore, OAT1 was the transporter most clearly responsive to HUA modeling and L-carnitine intervention in this study. Because OAT1 and OAT3 participate in basolateral urate and organic anion uptake, whereas URAT1 and GLUT9 are mainly involved in urate reabsorption, OAT1 restoration may indicate an OAT1-related transporter response potentially relevant to renal secretory handling. Nevertheless, the involvement of other renal and intestinal urate transporters cannot be excluded, and direct renal urate excretory function cannot be inferred without urinary uric acid excretion or fractional urate excretion measurements (8, 9, 36).

The URAT1 findings should also be interpreted cautiously. L-carnitine did not consistently suppress URAT1, suggesting that inhibition of URAT1-dependent urate reabsorption was unlikely to be its predominant renal response. Allopurinol markedly reduced serum UA, confirming the responsiveness of the HUA model to a classical urate-lowering intervention. However, because allopurinol primarily inhibits xanthine oxidase-mediated urate production, it is not a mechanism-matched comparator for renal or intestinal urate excretion. Accordingly, the increased URAT1 expression and elevated serum urea observed in the allopurinol group were regarded as treatment-associated or adaptive responses rather than evidence of uricosuric activity (37–39).

Renal transcriptomic analysis provided additional context for the renal effects of L-carnitine. Among the enriched pathways, NF-κB signaling was selected for further validation because of its established association with renal inflammation and tubular injury under hyperuricemic conditions (40, 41). The increased renal p-p65/p65 ratio, reduced IκBα expression, and enhanced nuclear accumulation of p65 in HUA mice were attenuated by L-carnitine treatment. These findings support an association between L-carnitine supplementation and reduced renal NF-κB-related inflammatory signaling. Attenuation of NF-κB-related signaling co-occurred with recovery of OAT1 expression; however, the present data do not establish a causal relationship between these two responses. Other pathways identified by transcriptomic enrichment, including PI3K-Akt, calcium signaling, and Wnt-related pathways, remain exploratory findings and require independent validation.

The intestinal findings further indicate that the response to L-carnitine was not confined to the kidney. Hyperuricemic mice exhibited mild villus abnormalities and reduced expression of the tight-junction proteins ZO-1 and Occludin, whereas L-carnitine improved these barrier-related changes and increased intestinal ABCG2 expression. ABCG2 contributes to extra-renal intestinal urate transport and represents an important component of intestinal urate handling (11, 42). In addition, high-dose L-carnitine significantly increased endpoint fecal UA content compared with untreated HUA mice. This finding provides an additional intestinal-side observation associated with the urate-lowering response. Nevertheless, because feces were not collected over a standardized 24-h period and total fecal output was not recorded, endpoint fecal UA content cannot be equated with quantitative fecal urate excretion. Thus, the ABCG2 and fecal UA findings support an intestinal urate-handling-related response but do not demonstrate enhanced intestinal urate excretion.

Gut microbiota changes may provide additional context for the concurrent intestinal and renal responses. Previous studies have linked microbiota dysbiosis in hyperuricemia with altered intestinal urate metabolism, epithelial barrier dysfunction, and renal inflammatory signaling, while microbial metabolites such as short-chain fatty acids may contribute to maintenance of intestinal barrier integrity (43–45). In the present study, high-dose L-carnitine partially restored microbial diversity and community structure. Together with the changes in intestinal barrier proteins, ABCG2, renal OAT1, and NF-κB-related signaling, these findings are compatible with a gut–kidney axis framework. However, the 16S rRNA analysis included only the CG, HUG, and HLG groups and four biological replicates per group, and no microbial metabolite analysis or functional microbiota intervention was performed. Therefore, the microbiota findings should be regarded as exploratory community-level associations rather than evidence that microbiota remodeling mediates the urate-lowering effect of L-carnitine.

The potential translational relevance of L-carnitine should also be considered together with its safety profile. Although the doses used in the present study produced beneficial effects without obvious hepatic histopathological injury, previous studies have raised concerns regarding possible hepatic oxidative stress, inflammatory responses, and altered organic ion transport following prolonged or high-dose supplementation (46). Moreover, gut microbiota-mediated conversion of L-carnitine to trimethylamine and trimethylamine-N-oxide may be particularly relevant during long-term administration and in individuals with impaired renal function (47, 48). Therefore, the present findings should not be interpreted as evidence supporting routine clinical use of L-carnitine for hyperuricemia. Its long-term safety, dose–response relationship, efficacy, and appropriate target population require further investigation.

Several limitations should be acknowledged. First, rodents retain uricase activity and therefore do not fully reproduce human urate metabolism. Second, although seven animals per group were included in the main *in vivo* efficacy experiment, several molecular assays used only three or four biological replicates, and renal RNA-seq and 16S rRNA sequencing included only CG, HUG, and HLG. Consequently, these exploratory datasets cannot establish dose–response relationships or clearly distinguish L-carnitine-specific responses from general treatment-associated effects. Third, direct urate excretion was not quantified. Metabolic cage-based 24-h urine and feces collection was not performed; therefore, urinary creatinine, urate clearance, fractional urate excretion, and total fecal urate excretion could not be determined. Transporter functional assays and membrane-localization analyses were also not performed. Thus, changes in OAT1, intestinal ABCG2, and endpoint fecal UA content remain correlative evidence of altered urate handling. Fourth, allopurinol served as a model-responsiveness reference rather than a mechanism-matched uricosuric comparator; future studies should include an appropriate uricosuric control. Fifth, although NF-κB-related changes were supported by protein and immunofluorescence analyses, inhibitor, gene-silencing, or other functional experiments are required to establish causality, whereas other transcriptomically enriched pathways, including PI3K-Akt signaling, remain unvalidated. Finally, the microbiota findings require confirmation using larger cohorts and functional approaches such as metabolite profiling, antibiotic intervention, or fecal microbiota transplantation.

## 5 Conclusion

Oral L-carnitine supplementation reduced serum uric acid levels in hyperuricemic mice and was associated with renal and intestinal urate-handling-related responses. This effect was not accompanied by detectable suppression of hepatic urate production, but was associated with recovery of renal OAT1 expression, intestinal barrier-related improvement, increased intestinal ABCG2 expression, attenuation of renal inflammatory signaling, and partial restoration of gut microbiota structure. These findings support oral L-carnitine as a potential nutritional modulator of urate homeostasis. However, enhanced renal or intestinal urate excretion was not demonstrated, and direct functional evidence remains to be established.

## Data Availability

The raw data analyzed in this study are available in the NCBI Sequence Read Archive (https://www.ncbi.nlm.nih.gov/sra) under accession numbers PRJNA1522548 and PRJNA1522495.
